# Circadian, Reward, and Emotion Systems in Teens prospective longitudinal study: protocol overview of an integrative reward-circadian rhythm model of first onset of bipolar spectrum disorder in adolescence

**DOI:** 10.1186/s12888-023-05094-z

**Published:** 2023-08-17

**Authors:** Lauren B. Alloy, Rachel F. L. Walsh, Logan T. Smith, Mackenzie A. Maddox, Thomas M. Olino, Phyllis C. Zee, Robin Nusslock

**Affiliations:** 1 Department of Psychology and Neuroscience, Temple University, Philadelphia, USA; 2 Department of Neurology, Feinberg School of Medicine, Northwestern University, Evanston, USA; 3 Department of Psychology, Northwestern University, Evanston, USA

**Keywords:** Bipolar disorder, Hypo/mania, Depression, Reward responsiveness, Circadian rhythms, fMRI, Stress, Adversity, Sleep, Ecological momentary assessment

## Abstract

**Background:**

Bipolar spectrum disorders (BSDs) are associated with a heightened sensitivity to rewards and elevated reward-related brain function in cortico-striatal circuitry. A separate literature documents social and circadian rhythm disruption in BSDs. Recently, integrated reward-circadian models of BSDs have been proposed. These models draw on work indicating that the two systems influence each other and interact to affect mood functioning. When dysregulated, reward and circadian system signaling may combine to form a positive feedback loop, whereby dysregulation in one system exacerbates dysregulation in the other. Project CREST (Circadian, Reward, and Emotion Systems in Teens) provides a first systematic test of reward-circadian dysregulation as a synergistic and dynamic vulnerability for first onset of BSD and increases in bipolar symptoms during adolescence.

**Methods:**

This NIMH-funded R01 study is a 3-year prospective, longitudinal investigation of approximately 320 community adolescents from the broader Philadelphia area, United States of America. Eligible participants must be 13–16 years old, fluent in English, and without a prior BSD or hypomanic episode. They are being selected along the entire dimension of self-reported reward responsiveness, with oversampling at the high tail of the dimension in order to increase the likelihood of BSD onsets. At Times 1–6, every 6 months, participants will complete assessments of reward-relevant and social rhythm disruption life events and self-report and diagnostic assessments of bipolar symptoms and episodes. Yearly, at Times 1, 3, and 5, participants also will complete self-report measures of circadian chronotype (morningness-eveningness) and social rhythm regularity, a salivary dim light melatonin onset (DLMO) procedure to assess circadian phase, self-report, behavioral, and neural (fMRI) assessments of monetary and social reward responsiveness, and a 7-day ecological momentary assessment (EMA) period. During each EMA period, participants will complete continuous measures of sleep/wake and activity (actigraphy), a daily sleep diary, and three within-day (morning, afternoon, evening) measures of life events coded for reward-relevance and social rhythm disruption, monetary and social reward responsiveness, positive and negative affect, and hypo/manic and depressive symptoms. The fMRI scan will occur on the day before and the DLMO procedure will occur on the first evening of the 7-day EMA period.

**Discussion:**

This study is an innovative integration of research on multi-organ systems involved in reward and circadian signaling in understanding first onset of BSD in adolescence. It has the potential to facilitate novel pharmacological, neural, and behavioral interventions to treat, and ideally prevent, bipolar conditions.

**Supplementary Information:**

The online version contains supplementary material available at 10.1186/s12888-023-05094-z.

## Background

Bipolar spectrum disorders (BSDs) are prevalent, highly recurrent, and a major public health problem accounting for much disability, mortality, and poor quality of life worldwide [1, 2]. BSDs form a spectrum of severity from the milder cyclothymia, to bipolar II, to full blown bipolar I disorder [3, 4]. Even subsyndromal symptoms of BSDs are associated with significant functional impairment and increased suicide risk [5, 6], and the milder forms of BSD often progress to more severe disorders over time [3, 7]. Adolescence is an “age of risk” for the emergence of bipolar symptoms and first onset of BSD [8, 9]. However, the mechanisms responsible for adolescent vulnerability to BSD are not well understood. Yet, knowledge about vulnerability mechanisms is crucial for understanding etiological pathways to BSD and for translating foundational knowledge about the risk factors and causes of BSDs to powerful, theoretically coherent interventions that prevent or treat these often-impairing conditions. Thus, the overarching goal of Project CREST (Circadian, Reward, and Emotion Systems in Teens; R01MH126911) is to test a novel, integrated reward-circadian rhythm dysregulation approach to increases in bipolar symptoms and first onset of BSD in the vulnerable period of adolescence.

### Circadian and social rhythm disruption and risk for BSDs

Biological processes that repeat about every 24 h and persist with the same period in the absence of external cues are defined as circadian rhythms [10]. The suprachiasmatic nucleus (SCN), or “biological clock”, regulates circadian rhythms [10, 11], and is highly sensitive to exogenous cues [12]. Circadian rhythms normally are entrained or phase aligned to the physical environment predominately by the external zeitgeber (“time giver”), light [12, 13], but also by nonphotic cues, including daily schedules or social rhythms [14, 15]. Circadian models of BSDs propose that circadian dysregulation leads to bipolar hypomanic and manic (hereafter referred to as hypo/manic) and depressive symptoms and episodes and provides vulnerability to BSD onset [16–19]. Many individuals with BSDs have circadian rhythm alterations of sleep-wake, activity, melatonin, and other hormones even in euthymic periods. Individuals with hypo/manic symptoms also show activity rhythm irregularities as measured by actigraphy and self-report. In addition, individuals with BSDs exhibit clock gene variants that show irregular rhythm patterns [16, 20]. However, not all studies uniformly support a role for circadian dysregulation in BSDs [21–23]. And, an important limitation of prior research is that it has never tested whether circadian rhythm dysregulation provides vulnerability to first onset of BSD prospectively.

**Fig. 1:**
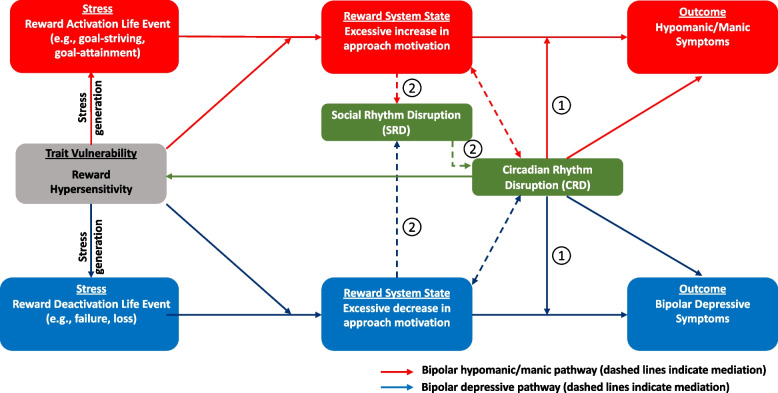
Integrated reward-circadian model of bipolar spectrum disorders

According to the social zeitgeber theory [17, 18, 24], life events that disrupt daily social rhythms or schedules precipitate bipolar symptoms by disturbing circadian rhythms (green boxes in Fig. 1). Social rhythm disruption (SRD) events may lead to changes in light exposure, which can phase shift melatonin and other circadian rhythms [12, 13, 25], or may disrupt circadian rhythms through their effects on non-photic zeitgebers [17, 18, 26]. Indeed, individuals with BSDs exhibit less social rhythm regularity than controls [27–29], and this irregularity predicts greater mood symptom variability [30] and shorter time to recurrence of bipolar depressive and hypo/manic episodes [29]. The efficacy of treatments involving social rhythm stabilization for patients with BSDs also supports the role of SRD in bipolar disorder [31, 32]. In people with BSD, SRD events were more likely to occur prior to manic or depressive episodes relative to control periods [33–35], and an increase in SRD events predicted a shorter time to bipolar depressive episode recurrence [35]. Individuals with BSD also were more susceptible than controls to SRD in response to equivalent life events [36, 37]. Although baseline social rhythm regularity and SRD events predict bipolar symptoms and episodes [33–35], whether this is due to circadian rhythm disruption has not been demonstrated. Repeated assessments of social, behavioral, and circadian rhythms in ambulatory participants over time, allowing for temporal precedence, stronger causal inferences, and examination of trajectories during adolescent development, are needed to test whether SRD and circadian rhythm disruption predict first onset of BSD and bipolar symptoms in adolescents’ daily lives. Project CREST is designed to provide such novel evidence.

### Reward hypersensitivity and risk for BSDs

In a separate literature, BSDs have been associated with an enhanced sensitivity to rewarding stimuli and higher reward-related brain function in cortico-striatal circuitry [9, 38, 39]. Moreover, high reward responsiveness (RR) is hypothesized to be a vulnerability for BSDs [9, 38–40]. According to these reward hypersensitivity models of BSDs, high trait RR can lead to excessive state increases in approach-related affect and behavior in response to life events that activate the reward system involving goal-striving or attainment, which in the extreme, is reflected in hypomanic symptoms or episodes (Fig. 1, red pathway). It also can lead to excessive state decreases in approach-related affect and behavior in response to events that deactivate the reward system involving definite failures and losses, reflected in bipolar depressive symptoms or episodes (Fig. 1, blue pathway). Reward hypersensitivity should make people hyper-reactive to cues signaling both the possible attainment and loss of reward, thus increasing risk for both hypo/mania and bipolar depression (vulnerability-stress hypothesis). From this perspective, mixed states may reflect rapid oscillations between increases and decreases in approach motivation in response to reward-activation and -deactivation cues, respectively, within the same day, leading to rapid switches between hypo/mania and depression that appear simultaneous in retrospective recall [9]. Alternatively, mixed states may involve the co-occurrence of reward-activation and –deactivation cues leading to a mixed motivational state and both hypo/manic and depressive symptoms simultaneously.

Much evidence supports the reward hypersensitivity model of BSDs (see [9, 38–40] for reviews). Individuals with or at risk for BSDs exhibit ambitious goal-striving [41–44], and greater behavioral, emotional, cognitive, and neurophysiological responses to rewards than controls [8, 45–49]. High self-report, behavioral, and/or neurophysiological RR predicts first onset and recurrences of BSD [8, 50], and progression to more severe bipolar diagnoses (e.g., bipolar I) among individuals with milder BSDs [7]. Further, reward-activation events involving goal-striving or attainment predict hypo/manic episodes among BSD participants [51–53], whereas reward-deactivation events predict depressive episodes [54–56]. Finally, fMRI studies provide partial support for elevated ventral striatum (VS) and orbitofrontal cortex (OFC) reward responses in manic and euthymic bipolar participants and participants at risk for BSD (see [39] for review, but see [57–60] for contrary results). Moreover, individuals at risk for BSD show greater cortico-striatal structural connectivity than low-risk participants [61] and those with a BSD exhibit greater gray matter volume in structures of the reward circuitry [62].

To test the reward hypersensitivity theory of vulnerability for BSD, a truly prospective, longitudinal study of first onset of BSD is required. Although a few studies examined baseline RR as a predictor of bipolar symptoms or episodes [7, 49, 50, 63], whether chronically high RR and increases in RR during adolescence predicts first onset of BSD is untested. This is important because the neural circuitry underlying reward function shows rapid maturation during adolescence [64, 65]. At least three timepoints are required to test whether developmental trajectories of RR predict first onset of BSD and increases in bipolar symptoms, as well as to test potential mediators of the RR – bipolar symptoms and BSD onset predictive associations. In addition, almost all research on the role of reward function in BSD has been limited to financial incentives. Given that social rewards increase in salience during adolescence [66–68], we also examine the trajectories of social reward processing as a predictor of first onset of BSD and increases in bipolar symptoms.

### Integrated reward and circadian rhythm model of BSD

Furthermore, work on circadian and reward approaches to BSDs mostly has proceeded in parallel. Recently, however, integrated reward-circadian models of BSDs have been proposed [24, 69] based on evidence that the SCN and cortico-striatal reward circuitry are related bidirectionally [26, 69–73] and interact to affect mood processes [26, 55]. Indeed, RR exhibits time-of-day and circadian activation effects [70, 71, 74], clock genes are expressed in brain reward areas [75–77], and midbrain dopamine neurons project directly to the rodent SCN [78]. When regulated properly, reward-circadian interactions coordinate adaptive mood and behavioral functioning for goal pursuit. However, when either system becomes dysregulated, reward and circadian signaling may synergize to form a positive feedback loop, whereby dysregulation in one system exacerbates dysregulation in the other. Under these conditions, reward and circadian dysfunctions may interact to predict maladaptive mood and behavior, as reflected in hypo/manic and depressive symptoms (moderation hypothesis; arrows labeled “1” in Fig. 1). However, no prior study has tested whether RR interacts with circadian rhythm disruption to predict BSD onset and symptoms prospectively.

#### Reward-to-circadian pathway

According to the Reward Circadian Rhythm (RCR) model of BSDs [24], in vulnerable reward-hypersensitive individuals, events that activate (e.g., goal-striving and attainment) or deactivate (e.g., goal failures and losses) the reward system lead to excessive behavioral and neural states of reward activation or deactivation, respectively. In turn, this is hypothesized to indirectly (through behaviors that generate SRD events) lead to circadian rhythm disruption (mediation hypothesis; arrows labeled “2” and green boxes, Fig. 1). Via this indirect pathway through SRD events, when reward hypersensitive individuals experience excessive reward activation in response to goals or rewards, they should exhibit excessively high goal-striving and response initiation, incongruent with maintaining regular social rhythms [24, 36, 55, 79]. Thus, they may work excessively long hours and neglect normal social routines, which, in turn, may disrupt circadian rhythms and trigger hypo/manic symptoms [24, 55, 79]. Similarly, reward-deactivation events can lead to excessively decreased approach and response initiation and disregard of social routines, and lead to BSD depressive symptoms. Yet, research is needed to test whether SRD or circadian rhythm disruption mediates the interaction of RR and reward-relevant life events in predicting BSD onset and symptoms, as proposed in the RCR model.

#### Circadian-to-reward pathway

In the RCR model, circadian rhythms also influence RR (solid green feedback path, Fig. 1). Reward system activation may be modulated by SCN timing information as discussed above. Indeed, circadian influence on RR is well documented in animals, with the strongest evidence for dopamine-mediated reward motivation [80]. In humans, reward motivation also shows circadian influences [69], positive affect exhibits a circadian rhythm [69, 72, 81] and cortico-striatal RR shows time-of-day effects [70]. However, no study has tested whether circadian rhythm disruption predicts BSD symptoms via mediation by RR.

### The present study

Project CREST provides the first systematic test of reward-circadian dysregulation as a joint vulnerability for increases in bipolar symptoms and first onset of BSD in the vulnerable period of adolescence. We use a biobehavioral high-risk design involving multilevel social and circadian rhythm measures (self-report, actigraphy, melatonin) and multilevel (self-report, behavioral, neural) and multimodal (monetary, social) RR measures in a prospective longitudinal design with repeated ecological momentary assessment (EMA) periods to examine: 1) concurrent and longitudinal bidirectional associations between social and circadian rhythm disruption and RR; 2) mediators of their associations, and 3) social and circadian rhythm dysregulation and RR as separate and joint predictors of increases in BSD symptoms and risk for first onset of BSD during adolescence.

Our hypotheses are as follows: 1) We predict that higher RR will be associated with greater social and circadian rhythm dysregulation (phase shift, lower amplitude, and less regular rhythms) concurrently and that increases in RR over time will predict greater social and circadian rhythm dysregulation over time and vice versa. 2) During EMA periods: a) reward-activation and -deactivation events will predict hypo/manic and depressive symptoms, respectively, mediated by SRD and circadian rhythm disruption; b) between-day changes in social and circadian rhythm disruption will predict hypo/manic and depressive symptoms, mediated by RR; and c) greater mood symptoms via these pathways will occur for participants selected for high trait RR. 3) Finally, we also predict that chronically high and increasing trajectories of RR and chronically high and worsening trajectories of social and circadian rhythm dysregulation will be separate and joint (interactive) predictors of increases in hypo/manic and depressive symptoms and first onset of BSD over time.

The goal is to recruit 320, 13–16 year-old adolescents to complete a prospective, 3-year longitudinal study. Recruitment began in Spring 2022. This age range was chosen based on several considerations. First, epidemiological studies of BSD incidence suggest that the first peak in BSD onset occurs between ages 14–19 [9, 82, 83]; thus, we wanted to recruit participants in the first half of this age range and follow them through most of this risk period. Second, RR normatively increases in adolescence and may start to peak around ages 15–16 [64, 65]. Third, sleep and circadian rhythms change normatively in adolescence [84–86]. Thus, this age range should provide the best opportunity to observe trajectories of RR-circadian rhythm associations and prediction of first onset of BSD.

**Fig. 2:**
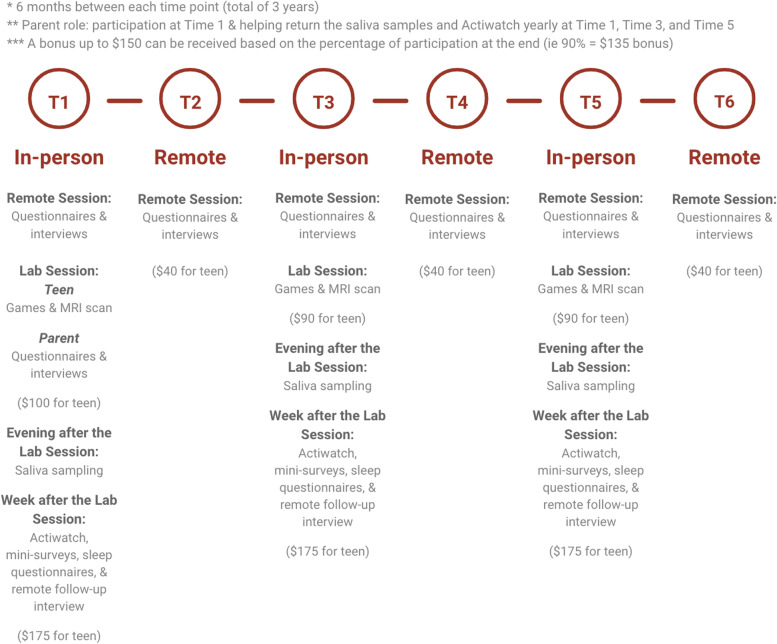
Study timeline

Participants with no prior history of BSD are being selected along the entire dimension of self-reported trait RR, with oversampling at the high tail of the dimension to increase the likelihood of BSD onsets [8]. At Time 1 (T1) – Time 6 (T6), every 6 months, participants will complete self-report and interview assessments of life events coded for SRD and reward-relevance and self-report and psychiatric diagnostic interview measures of hypomanic and depressive symptoms and episodes for a total of 3 years of follow-up. Yearly, at T1, T3, and T5, participants also will complete self-report measures of circadian chronotype and social rhythm regularity, dim light melatonin onset (DLMO) saliva sampling procedures to assess circadian phase, self-report RR, goal-striving, and ruminative response styles, behavioral and neural RR (fMRI assessment of cortico-striatal [VS, OFC)] activation and functional connectivity during monetary and social reward tasks), and a 7-day EMA period. The behavioral and fMRI RR measures will occur on the day before the DLMO procedure and the start of the 7-day EMA period. During each 7-day EMA period, participants will complete continuous measures of sleep/wake and activity (actigraphy) and 3 within-day (morning, afternoon, evening) measures of life events coded for SRD and reward-relevance, monetary and social RR ratings, positive and negative affect, and hypo/manic and depressive symptoms. At T1 only, mothers also will provide information on their own psychiatric history, family psychiatric history, socioeconomic status (SES), and adolescent participants’ adversity history from birth to T1 (see Fig. 2). A STROBE Checklist [87] for this study may be found in Supplementary Materials.

## Methods

### Participant recruitment, eligibility, and characteristics

A two-phase screening process is used to recruit adolescents from the community. First, they complete an online screening questionnaire including demographics questions, the Behavioral Inhibition System/Behavioral Activation System (BIS/BAS) [88] Scales, and contact information for themselves and a parent. The BAS total score from the BIS/BAS is a psychometrically reliable and valid measure of trait reward sensitivity [7, 8, 89] that we have used before to select participants with different levels of trait RR [8]. Based on their BAS scores, we plan to recruit 20 adolescents each from the 0-20^th^, 21-40^th^, 41-60^th^, and 61-80^th^ quintiles, and 240 adolescents from the highest 80-100^th^ quintile of the RR dimension. The purpose of this recruitment design involving oversampling of participants in the highest quintile of BAS scores is to ensure that we have enough participants at elevated risk for first onset of BSD, while still maintaining representation of the full dimension of RR in the sample. To be eligible for the study, adolescents must be ages 13–16 and all racial and ethnic groups are eligible. To allow exploration of sex differences, we will try to recruit equal numbers of males and females.

Adolescents who are potentially eligible based on the online screener and their parents are then contacted to schedule a Phase II phone screening interview. The phone screening interview is used to describe the project to adolescents and their parents in detail and, if they are interested, to more fully determine whether the adolescent meets all eligibility criteria. The interview contains an MRI safety screening questionnaire, questions about eligibility for the circadian component of the study, and the current and past mood disorder sections of the Structured Clinical Interview for DSM-5 (SCID-5) [90]. The original source of this description of the phone screening interview may be found in Alloy et al. [91]. Eligible adolescents who wish to participate provide written assent and their parent provides written consent. Project CREST is approved by the Temple University IRB (#28338) and is being conducted in accordance with all relevant guidelines and regulations, including the Declaration of Helsinki.

We exclude adolescents with a history of a BSD (bipolar I, bipolar II, cyclothymia, or even a single hypomanic episode) because one of the aims of the project is to predict first onset of BSD in adolescence. To ensure validity of project assessments, we also exclude participants with psychotic symptoms (hallucinations, delusions). Standard MRI exclusion criteria (e.g., traumatic brain injury, pregnancy, metal in the body, severe claustrophobia) also are applied. In addition, adolescents are excluded based on the following: 1) use of melatonin or photosensitizing medications, 2) current or past treatment with bright light, and 3) shift work or travel across multiple time zones within the past month. These exclusion criteria are designed to ensure that there are no contraindications or major confounding factors for the DLMO saliva sampling procedure. If an adolescent has traveled across time zones, but is otherwise eligible, participation in T1 is delayed for at least 1 month. Psychotropic medication use is not a basis of exclusion; instead, analyses will control for medication use. This is designed to increase the sample’s representativeness and the generalizability of project findings, inasmuch as individuals with, and at risk for mood disorders often show elevated rates of psychotropic medication use [92].

### Statistical power estimates

Power analyses were conducted at the time of the NIMH grant application (MH126911) and prior to the start of data collection for Project CREST and informed sample size. All power estimates were derived using Monte Carlo simulations facilitated in Mplus 8.4 and were based on 1000 replications. For Hypothesis 1 cross-sectional associations, based on our planned sample size, we have strong power (> 0.90) to detect small (*r* = 0.20) bivariate associations. For longitudinal associations, we have good power (> 0.81) to detect small-moderate associations (*r* = 0.275) of prediction of slopes of one system from the intercept of the other within planned parallel process growth models.

For Hypothesis 2, within each EMA period, we will examine mediating relationships within the context of random-intercept cross-lagged panel (RICLP) models that include processes for each predictor, mediator, and outcome variable. Models focus on the indirect effects from the within-person assessments of the predictor to the outcome through the mediator variables and include complementary between-person associations to permit discriminating associations between levels of analysis. Within the context of these models, there is strong power (> 0.90) to detect small effects (*r* = 0.25) for between- and within-person associations. Further, power is strong (> 0.90) to detect significant indirect effects at both the within- and between-person levels of analysis. Moreover, we have good power (> 0.85) to detect small differences in the magnitude of indirect effects (i.e., standardized indirect effects differing by 0.06) in the context of the hypothesized interaction effects.

For Hypothesis 3, we have good power to detect small associations between growth factors of RR and social and circadian rhythm trajectories with symptom trajectories (> 0.85) and moderate power to detect moderate effects for prediction of BSD onsets (> 0.81). Based on findings from Alloy et al. [8], we estimate that 20.9% (*N* = 50) of the 240 participants from the highest quintile (80-100^th^ %) of the RR dimension, and 4.2% (*N* = 3) of the 80 participants from the rest of the RR dimension (0-79^th^ %), will develop a first onset of BSD by T6, for a total of 53 BSD onset cases. We also have adequate power (0.78) to detect moderate interaction effects (i.e., standardized regression coefficients differing by 0.30) predicting symptoms and similar power (0.82) for finding significant interaction effects for prediction of BSD onsets (i.e., odds-ratios for the interaction effect of approximately 1.6).

### Measures

Study assessments and their timing are summarized in Tables 1 and 2. Baseline T1 and yearly T3 and T5 involve two sessions designed to occur within a week of each other (Session 1 is remote over videoconferencing; Session 2 is in person) and T2, T4, and T6, occurring 6 months after T1, T3, and T5 involve one remote session via videoconferencing (see Fig. 2).

**Table 1:** Summary and timing of assessments

**Construct**	**T1** **S1**	**T1** **S2**	**T2**	**T3** **S1**	**T3** **S2**	**T4**	**T5** **S1**	**T5** **S2**	**T6**
**Reward Responsiveness (RR)**									
Behavioral Inhibition Scale/Behavioral Activation Scale (BIS/BAS)	✓			✓			✓		
Sensitivity to Punishment/Sensitivity to Reward Ques. (SPSRQ)	✓			✓			✓		
Positive Valence Systems Scale (PVSS)	✓			✓			✓		
Ambitious Goal-Striving Scale (WASSUP)	✓			✓			✓		
Card Arranging Reward Responsivity Objective Test (CARROT)		✓			✓			✓	
fMRI Monetary Incentive Delay Task (MID)		✓			✓			✓	
fMRI Chatroom Interact Task (CHAT)		✓			✓			✓	
Mock Scanner		✓			✓			✓	
**Social and Circadian Rhythms**									
Social Rhythm Metric-Trait (SRM-T)		✓			✓			✓	
Morningness-Eveningness Questionnaire (MEQ)	✓			✓			✓		
Dim Light Melatonin Onset (DLMO)		✓			✓			✓	
Pittsburgh Sleep Quality Index (PSQI)	✓			✓			✓		
7-Day EMA Periods (see Table 2 for details)		✓			✓			✓	
DLMO, Actigraphy, & EMA Instruction Session		✓			✓			✓	
**Childhood/Adolescent Adversity**									
Childhood Life Events Scale (CLES) (Ps & Mothers)	✓								
**Current and Prospective Life Events and Response Styles**									
Life Events Scale (LES) & Interview (LEI) (Reward & SRD coding)	✓		✓	✓		✓	✓		✓
Ruminative Response Styles (RRS)	✓			✓			✓		
Responses to Positive Affect Scale (RPAS)	✓			✓			✓		
**Diagnoses/Symptoms/Pubertal Maturation**									
Expanded SCID-5 Diagnostic Interview	✓		✓	✓		✓	✓		✓
Family History Interview (FHI) (Mothers)	✓								
Beck Depression Inventory - II (BDI-II)		✓	✓		✓	✓		✓	✓
Altman Self-Rating Mania Scale (ASRM)		✓	✓		✓	✓		✓	✓
Child Mania Rating Scale 10 – Self Report (CMRS-SR)		✓	✓		✓	✓		✓	✓
10 Mania 10 Depression Scales (10M10D)		✓	✓		✓	✓		✓	✓
Internal State Scale-Short Version (ISS-SV) – done on day of scan		✓			✓			✓	
Adolescent Alcohol and Drug Involvement Scale (AADIS)		✓	✓		✓	✓		✓	✓
Pubertal Development Scale (PDS)	✓		✓	✓		✓	✓	✓	✓
**Other**									
Child SES Interview – Parent Version (Mothers)	✓								
MacArthur Scale of Subjective Social Status (Ps and Mothers)	✓								
Chapman Handedness Scale	✓

**Table 2:** Assessments in 7-day ecological momentary assessment (EMA) periods

**Measures**	**1x/Day**	**3x/day**	**Continuous**	**End of 7-days**
**Life Events**				
Positive & Negative Events (Reward, SRD coding)		✓		
Life Events Interview about Events during 7 Days				✓
**Circadian Rhythms/Sleep/Activity Parameters**				
Actigraphy (Sleep/wake & activity patterns)			✓	
Sleep Diary (before bed)	✓			
**Mood and Symptoms**				
Positive & Negative Affect Scale (4 PA, 4 NA items)		✓		
Depressive Symptoms (5 BDI items)		✓		
Hypomanic/Manic Symptoms (5 ASRM items)		✓		
**Reward Responsivity (RR)**				
Reward Ratings (3 monetary & 3 social items)		✓		

### Reward measures

The self-report, behavioral, and neural reward measures are given yearly at T1, T3, and T5.

#### Self-report reward measures

In addition to the BAS subscale of the BIS/BAS [88], the Sensitivity to Reward (SR) subscale of the Sensitivity to Punishment (SP)/ Sensitivity to Reward (SR) Questionnaire (SPSRQ) [93] also is used to assess individual differences in trait sensitivity to social and non-social rewards [8]. The SPSRQ contains 48 yes-no items divided into SP and SR subscales. Subscale scores are derived by summing the number of “yes” responses on each scale. The SR subscale has shown strong retest reliability [93] and validity as a measure of trait reward sensitivity among adolescent samples [8].

Ambitious goal-striving is assessed with the Willingly Assumed Set of Statistically Unlikely Pursuits (WASSUP) [44]. Participants rate the likelihood of setting 30 very ambitious goals (social and non-social) for themselves. It is reliable, distinguishes individuals with mania from controls and high RR from moderate RR participants [41, 42, 44, 94], and predicts first onset of BSD [8].

The Positive Valence Systems Scale (PVSS) [95] contains 21 items that assess core constructs within the RDoC Positive Valence Systems domain (e.g., reward expectancy, reward anticipation) using seven reward types (i.e., food, physical touch, outdoors, positive feedback, hobbies, social interactions, and goals). Participants rate the extent to which the items describe their responses over the past two weeks on a 9-point Likert scale, ranging from 1 (*Extremely untrue of me*) to 9 (*Extremely true of me*). In adult samples, the PVSS demonstrated good retest reliability and internal consistency [95].

#### Behavioral reward measure

The Card Arranging Reward Responsivity Objective Test (CARROT) [96, 97] is a brief, three-trial, task measuring the extent to which individuals increase their card-sorting speed when offered small financial incentives compared to a no-reward condition. In this task, participants sort 60 cards into three numbered trays corresponding to whether the digits printed on the card include a 1, 2, or 3. Trial 1 is used to establish sorting speed. In Trial 2, participants are given 75% of their Trial 1 sorting time to sort another deck of cards. In Trial 3, they are given the same time limit, but the experimenter places a quarter in front of them after every 5 cards sorted. The RR measure is the number of cards sorted in Trial 3 minus Trial 2. The CARROT has been validated [48], correlates with self-reported RR [8, 98], and relates to the DRD2 dopamine gene [99]. The original source of the CARROT description may be found in Alloy et al. [91].

#### Neural reward measures

To examine neural (fMRI) responses to anticipation and receipt of monetary reward and loss, participants will complete the Monetary Incentive Delay task (MID; [100, 101]). On each trial, a circle cue is presented for 200 ms either indicating that participants can win or avoid losing money if they respond to a target stimulus in time (press a button before a solid white triangle disappears). The MID task consists of two runs, with each of the six types of trials presented eight times in random order. On each Win trial, participants can win $0.00, $1.50, or $5.00, and on each Loss trial, they can avoid losing $0.00, $1.50, and $5.00. On the Win trials, participants win money if they hit the white triangle in time and do not win money if they miss the target. On the Loss trials, they avoid losing money if they hit the white triangle in time and lose money if they miss it. Feedback about the amount of money won or lost then is displayed for 200 ms. The original source of this description of the MID task may be found in Alloy et al. [91]. The initial target duration for the MID task is determined based on each participant’s mean hit reaction time (ms) to the white triangle in a pre-scan task. As the task progresses, the target duration adapts in response to the previous trials to maintain task difficulty, such that each participant maintains an approximate 66% success rate. The analyses will focus on the reward anticipation phase, as defined by the time window between the offset of a circle cue and onset of a white triangle (target stimulus; 200–250 ms), and the reward outcome phase, as defined by the time window between the onset and the offset of the feedback (200 ms). The original source of this description may be found in Alloy et al. [91].

To examine neural (fMRI) responses to social rewards and losses, participants complete the Chatroom Interact Task [102–104]. It assesses reactions to social acceptance (i.e., reward) and rejection (i.e., loss) from virtual peers in an online setting. Participants are told that they will interact online with peers while in the scanner. Prior to engaging in the task in the scanner, participants provide their biographical profile and their photograph is taken. They also are shown fictitious biographical profiles and photographs of potential virtual peers they can choose to chat with. Participants are asked to select five same-sex peers that they would like to interact with during the task. Once in the scanner, the participant is told that they were matched with two same-sex peers who also are participating at different research sites. Participants review the photograph and biographical profile of the matched peers prior to the task. During the scan, pictures of the participant and the two virtual peers are projected onto the screen two at a time, and the participant and peers each take turns selecting who they would rather chat with about a series of interests (e.g., school, music, sports). The original source of this description of the Chatroom Interact task may be found in Alloy et al. [91]. The Chatroom task includes three experimental blocks, each with 15 trials, and a fourth control block (total run time 13 min, 30 s). In each experimental block, the participant or the peers are either chosen or not chosen to discuss a given topic. Stimuli are presented using Matlab (Version 9.10.0 [R2021a]; Mathworks). Each block begins with instruction about who will be selecting in that block (i.e., agent). The agent’s photo is displayed in the bottom left corner of the screen and the photos of the other two players are displayed in the middle of the screen. At the beginning of each trial, the question ‘who would you rather talk to about …’ with a selected topic for that trial (e.g. … ‘music?’) appears on the screen for 3.34 s (task component durations are chosen to be multiples of the 1.67 TR). Then, feedback is provided about the person who is chosen, indicated by a highlighted grey border around their photo, and the person who is not chosen, indicated by a superimposed gray ‘X’ on their photo. This feedback is presented for 10.02 s (i.e., 6 TR). The participant is instructed to press their index finger or middle finger to indicate whether the person on the left or right was chosen. The original source of this description may be found in Alloy et al. [91]. Trials are arranged in blocks so the participant experiences an ‘accept’ block, where they are chosen two thirds of the time, and a ‘reject’ block, where they are rejected two thirds of the time. Each block consists of the same topics (presented randomly), but with a different “agent.” In block 1, the participant is the “agent” and makes selections between the two same-gender peers. In blocks two and three, the participant is either chosen or not chosen by their virtual peers. Analyses are derived from these two blocks of ‘acceptance’ or ‘rejection’, with the order of accept versus reject trials randomized for each gender grouping. The fourth block is a perceptual and motor control task, where the picture of the participant and one virtual peer are displayed on the screen and a small grey dot appears on one of the faces. The participant is instructed to press their index finger or middle finger to indicate whether the person on the left or right has the dot. This block is designed to control for viewing faces (self and other) and pressing a button to identify a stimulus appearing on one of the faces. The original source of this description of the Chatroom Interact task may be found in Alloy et al. [91].

At the end of the Chatroom task, participants complete a debriefing questionnaire in which they rate their interest in the task and how happy, sad, angry, nervous, included, and excluded they felt when they were chosen and not chosen. They also are asked about how much experience they have with chatting online.

#### fMRI data acquisition, preprocessing, and analysis

A Prisma 3.0 Tesla Siemens MAGNETOM MRI scanner with a 64-channel gradient head coil is used to acquire fMRI data at Temple University. Prior to the scans, participants are trained on the fMRI procedures via mock scans. Functional runs use a slice-accelerated multiband EPI sequence (multiband acceleration factor: 2. GRAPPA acceleration factor: 2) covering 64 axial slices (voxel size = 2.0 × 2.0 × 2.0 mm; TR = 2050 ms; TE = 25 ms; FOV = 208 × 208 mm; Matrix = 104 × 104; Flip Angle 76°). Structural images are acquired using an MPRAGE sequence to obtain 208 axial slices (voxel size = 0.8 × 0.8 × 0.8 mm; TR = 2300 ms; TE = 2.99 ms; FOV = 256 × 256; Matrix = 320 × 320; Flip Angle = 7°). FIRMM software is used to generate real-time metrics of head motion, so we can give adolescents in-scanner feedback [105]. Data are processed using fMRIPrep [106, 107]. The original source of the description of the fMRI data acquisition, preprocessing, and analysis methods may be found in Alloy et al. [91].

For the MID Task, hemodynamic signal is deconvolved using a generalized linear model identifying six trial types (Win or Lose $0.00, $1.50, $5.00) during the anticipation and outcome phases. First-level voxel-wise t-statistics are computed for each participant contrasting reward (i.e., Win $1.50 and $5.00) vs. non-reward (i.e., Win $0.00) trials to calculate reward anticipation and outcome, and loss (i.e., Lose $1.50 and $5.00) vs. non-loss (i.e., Lose $0.00) trials to calculate loss anticipation and outcome [101, 108]. The analyses will include a nuisance regressor for high motion volumes (> 0.2 mm) and 6 motion parameters. The original source of the MID task analysis methods may be found in Alloy et al. [91].

For the Chatroom Interact Task, a first-level fixed-effect model will be constructed for each participant and predetermined conditioned effects at each voxel will be calculated using a t-statistic. Analyses will focus on reward trials (i.e., peer acceptance) versus the motor control task. Secondary analyses will examine loss trials (i.e., peer rejection) versus the motor control task to examine specificity of results to reward processing. Exploratory analyses will examine anticipation of reward. The original source of this analysis approach for the Chatroom task may be found in Alloy et al. [91].

To ensure independence from the functional data, we will use prior meta-analytic findings or anatomical atlases to define the a priori regions of interest (ROIs), ventral striatum (VS), bilateral orbitofrontal cortex (OFC), and ventromedial prefrontal cortex (vmPFC). To examine functional connectivity between the VS, OFC, vmPFC and/or other reward-relevant brain regions, we will use psychophysiological interaction (PPI) analysis. After generating the parameter estimates (beta-weights) of activation during reward anticipation and outcome in the ROIs, as well as PPI functional connectivity within the cortico-striatal circuit, the extracted parameter estimates will be imported into R statistical software for ROI activation and connectivity analyses. The original source of this description may be found in Alloy et al. [91].

### Social and circadian rhythm and sleep measures

Participants complete a series of self-report and objective measures of social/circadian rhythms and sleep yearly at T1, T3, and T5. To assess social rhythm regularity, participants complete the Social Rhythm Metric-Trait (SRM-T) [109]. This self-report measure seeks to quantify patterns of daily social behavior by assessing the regularity of the timing of 15 daily activities (e.g., bedtime, mealtime, exercise, etc.). Activities completed within 45 min of their average time are considered “regular” and a higher overall score reflects greater social rhythm regularity [109].

Chronotype, a trait-based indicator of circadian preference, is assessed using the Morningness-Eveningness Questionnaire (MEQ) [110]. This self-report measure assesses the degree to which individuals prefer morning or evening activities. Higher scores indicate greater morningness. Based on responses, individuals can be categorized as morning types (“morning larks”), evening types (“night owls”) or neither. The MEQ demonstrates good reliability [111] and is correlated with physiological markers such as dim light melatonin onset time [112, 113].

To assess sleep quality, participants complete the Pittsburgh Sleep Quality Index (PSQI), a 19-item self-report instrument that asks about the timing of sleep as well as sleep-related problems (e.g., insomnia, nightmares, breathing difficulties) over the past month [114]. This self-report measure consists of seven dimensions: subjective sleep quality, sleep latency, sleep duration, sleep efficiency, sleep disturbances, use of sleep medication, and daytime dysfunction, as well as a total score that reflects overall sleep disturbance [114]. Psychometric investigations indicate the PSQI demonstrates strong reliability and validity across multiple samples, including a community sample of adolescents [115, 116]. The PSQI has been shown to discriminate between individuals with and without disorders associated with sleep disturbance [117].

The T1, T3, and T5 study assessments are split into two sessions, one week apart, and during the one-week interval participants complete one week of actigraphy, daily sleep diaries, and a dim-light melatonin onset (DLMO) procedure. Actigraphy is an objective, reliable, and valid method for assessing activity patterns, sleep/wake metrics, and light levels, with minimal restriction or interruption of normal routines or daily activities [118–121]. Actigraphy has been well-validated against polysomnography and sleep diaries among healthy sleepers and clinical samples [119, 122, 123].

To collect actigraphy data, participants continuously wear a Philips Actiwatch with light sensors for 7 days on their non-dominant hand, only removing it when it might get wet (e.g., bathing). Participants are instructed to keep their watch uncovered (e.g., roll their shirt sleeve) to maximize the accuracy of environmental light measurement. Data will be sampled in 1-min epochs and cleaned by trained research assistants using a standardized protocol in which study staff will visually scan the actograms to evaluate whether the rest intervals align with the light data and subjective sleep and make adjustments when there are clear inconsistencies. Sleep parameters will be extracted (total sleep time, sleep duration, wake after sleep onset [WASO], and midpoint of sleep using Philips’ proprietary algorithms. Four circadian rhythm rest-activity measures also will be derived from the actigraphy data: 1) acrophase (the time of peak activity level each day), 2) amplitude (the difference between the activity level acrophase and nadir each day), 3) sleep midpoint, and 4) social jetlag (the discrepancy between weekday and weekend sleep) [28, 124]. Social jetlag will be computed by measuring the difference in sleep midpoint on “free” days (e.g., weekend) and “work” days (e.g., school days; [125]).

Participants also fill out a sleep diary each evening to collect self-report information about the past night’s sleep and factors that may have impacted their sleep. Sleep diaries are considered the “gold standard” for subjectively assessing sleep [126] and sleep experts recommend using a sleep diary in concert with actigraphy to reduce uncertainty and collect additional relevant information [120]. The sleep diary includes questions assessing the time participants woke up that morning and the following characteristics of last night’s sleep: the time they got in bed, the time they tried to fall asleep, the time they actually fell asleep, and the number of times they woke up during the night. Subjective sleep disturbances are assessed by asking whether participants had trouble 1) falling asleep, 2) staying asleep, or 3) waking up too early and being unable to return to sleep. Participants report previous day medication usage, caffeine consumption (coffee, soda, energy drink, tea), and whether they napped (and if so, at what time and for how long). The sleep diary also includes a single item assessing the quality of sleep, “*How well did you sleep last night?”* and a question asking how sleepy or alert participants felt during the day. Participants respond to these items on a 10-point Likert scale.

Dim light melatonin onset (DLMO), the gold standard physiological marker of endogenous circadian function [127], is assessed on the first full day of the EMA week (i.e., the evening after the fMRI scan during the second session of T1, T3, and T5). The day of the DLMO procedure, participants must refrain from alcohol, bananas, chocolate, NSAIDs, beverages with artificial colors, and any caffeinated beverage after breakfast to avoid sample contamination. Thirty minutes before the start of sample collection, participants dim their indoor lights, use a smartphone application to ensure the ambient light level is less than 100 lx, and don light-attenuating glasses. Participants provide 11 saliva samples starting 6 h before their bedtime. Sampling frequency is one sample an hour for 3 h and then one sample every 30 min for 3.5 h such that the last sample is taken right before going to sleep. Participants eat dinner during the first 1-h intervals and are instructed not to consume food or water within 30 and 20 min of a sample, respectively. Passive drool saliva samples (at least 1 mL) are collected in cryovials using a saliva collection aid. The exact timing of the sampling is recorded in two places: on a physical sheet of paper and in the Salimetrics “OnTimePoint” smartphone application. Samples are stored in participants’ freezer until they are returned to the lab, where they are stored at -20 °C pending ELISA assay. DLMO is defined as the first interpolated point (derived from between 2 points) at 4.0 pg/ml on the rising curve of melatonin concentration and is an objective indicator of circadian phase [112, 128]. The minimum detectable limit of the assay is 0.5 pg/ml with intra- and inter-assay coefficients of variation of 6.8% and 7.3%, respectively.

### Life events, adversity, and SES measures

Participants complete a modified version of the Life Events Scale (LES) [56] at T1-T6 to assess positive and negative, major and minor life events during the past 6 months. The original 193-item LES was shortened for Project CREST so that it contained 156 events in multiple domains (e.g., school, peers, romantic interests, family, financial) relevant to adolescents, including events particularly relevant to today (e.g., “*Received a lot more positive attention in-person or on social media (e.g., more “likes” than usual),”* “*Tested positive for COVID-19”*). Events were coded a priori as reward-relevant or not and into specific reward-relevant categories with inter-rater reliabilities of α’s = 0.79-0.94: Goal-Striving, Goal Attainment/Reward, Goal Obstacle, and Goal Failure/Loss [129]. The original source of the description of the LES may be found in Alloy et al. [91].

Following completion of the LES, adolescents complete a Life Events Interview (LEI) [56, 130] to obtain further information about the events endorsed on the LES and date when they occurred. Trained post-baccalaureate staff and clinical psychology doctoral students, blind to all study measures, conduct the interviews with adolescents. The LEI uses manualized, event-specific definitional criteria and probes to maintain consistency, avoid double-counting of events, and reduce dating errors. Events not meeting definitional criteria are disqualified to reduce subjective reporting biases. The LEI also employs the “gold-standard” contextual threat method [130] to rate the events’ objective impact on a scale from 1 (*Mild*) to 4 (*Extreme)* and independence (e.g., death of a family member) vs. dependence (e.g., fight with a friend) on the participant’s behavior. Social rhythm disruption (SRD) incurred from each qualifying event also is rated based on the number of hours the participant’s normal bedtime or wake time is changed by the event’s occurrence [35, 36, 55]. These procedures have yielded excellent reliability and validity of event dating and ratings (κ = 0.76 -0.89) in our previous studies [35, 36, 55, 56, 129]. The original source of the description of the LEI may be found in Alloy et al. [91].

The Children’s Life Events Scale (CLES) [131] is given to the parent and adolescent at T1 to assess negative events that the adolescent may have experienced throughout childhood. The parent also completes the CLES because there may be events that occurred when the adolescent was too young to remember. The CLES consists of a 50-item checklist including moderate to major negative events across the following domains: peer difficulties (e.g., “*break up of serious romantic relationship*”), achievement-related events (e.g., “*academic failure*”*),* family difficulties (e.g., “*divorce of parents*”, “*serious financial difficulties of family*”), maltreatment (i.e., emotional, sexual, physical), death of family member or close friend, and additional events suggesting inadequacy (e.g., “*acquired a physical deformity*”). For each event endorsed, the parent or adolescent reports the corresponding age of the adolescent at the time the event occurred. Scores for the CLES range from 0 to 50, with higher scores indicating a higher number of negative events, representing greater early adversity. Additional subset scores are derived from the total number of events reported within each subset category, including negative emotional feedback (i.e., “*frequent teasing by peers*”, “*decrease in acceptance by peers*”), family deaths (e.g., “*death of a grandparent*”, “*death of a parent*”), achievement failures (e.g., “*academic failure*”, “*nonacademic failure*”), events suggesting inadequacy (e.g., “*acquired a physical deformity*”, “*needed special education services*”), and dependent events and independent events. The CLES has shown predictive validity and good internal consistency (α = 0.75; [18, 131]).

At T1, both the teen and the parent complete the MacArthur Scale of Subjective Social Status [132, 133] in which they independently rank themselves on a 10-step ladder that represents their perception of their family’s social standing in the society. Lower rungs on the ladder indicate lower perceived social status whereas higher rungs indicate higher perceived social status. In addition, at T1, the parent is administered a childhood socioeconomic interview, which includes family structure, past and current income, past and current housing, parent education and employment status, and family immigration history. Family socioeconomic disadvantage is operationalized by taking the sum of the following indicators during both the first 5 years of the teen’s life and total lifetime (1 = present; 0 = absent): family poverty (i.e., income-to-poverty ratio < 1.00) [134], unemployment, single-parent family structure, parent non-completion of high school degree or equivalent, and immigration status. The original source of the description of the MacArthur Scale may be found in Alloy et al. [91].

#### Response style measures

The Ruminative Response Scale (RRS) [135] is a self-report questionnaire given to participants at T1, T3, and T5 to assess responses to a sad or depressed mood. The RRS short version contains 10 items across two subscales, brooding and reflection, which are differentially predictive of depression. Brooding, or moody pondering, involves fretting over personal shortcomings whereas reflection involves an individual’s ability to use a resolution-oriented point of view when thinking back on one’s self, thoughts, and feelings. Responses to items are rated on a 4-point Likert scale ranging from 1 (*almost never respond in this way*) to 4 (*almost always respond in this way*) with higher scores indicating greater rumination. Both subscales exhibit good convergent and predictive validity [135, 136].

Participants also are administered the Responses to Positive Affect Scale (RPAS) [137], which is a self-report questionnaire used to assess ruminative responses to positive affective states. The RPAS is given at T1, T3, and T5 and consists of 17 items across the following subscales: dampening, emotion-focused rumination, and self-focused rumination. The dampening subscale captures a person’s tendency to mitigate positive affect and is correlated with a history of depression [138], whereas the emotion-focused and self-focused rumination subscales capture a person’s tendency to amplify positive affect and are correlated with risk for hypomania [137]. All items are rated on a 4-point Likert scale (*I almost never respond in this way* to *I almost always respond in this way*) and demonstrate good internal reliability (α’s = 0.72-0.76) [137].

### Diagnostic and symptom measures

#### Diagnostic interview measure

At T1, the Structured Clinical Interview for DSM-5 (SCID-5) [90] is used to assess lifetime history of mood and other disorders based on DSM-5 criteria [139]. Project CREST utilizes the Nonpatient Version Overview and Modules A (Mood Episodes), B/C (Psychotic Symptom Screen), D (Differential Diagnosis of Mood Disorders), E (Substance Use Disorders), F (Anxiety Disorders), G (Obsessive-Compulsive and Related Disorders), I (Feeding and Eating Disorders), K (Externalizing Disorders), and L (Trauma- and Stressor-Related Disorders) of the SCID-5 [90], as well as the expanded SADS-L (exp-SADS-L) diagnostic interview [8, 140] for current and past mood episodes and symptoms. These mood modules were combined to include DSM-5 criteria and avoid skip outs in the mood disorder sections, ensuring that we obtain all mood symptom ratings, even if a participant endorses a period of mood symptoms for a duration shorter than required by DSM-5 criteria. In addition, at T1, adolescents’ primary caregiver also completes a Family History Interview [141] to assess history of mood disorders for the adolescent participant’s 1^st^ and 2^nd^ degree relatives. Every 6-months (T2-T6) after T1, interviewer-rated symptoms, functioning, and onsets of DSM-5 mood and other disorders since the previous interview are assessed using this modified version of the SCID-5 with expanded mood modules and no skip outs in the mood modules. The original source of the description of the diagnostic interview may be found in Alloy et al. [91].

The expanded SCID-5 is administered to adolescents by trained post-baccalaureate and clinical psychology doctoral student interviewers, blind to participants’ reward quintile. Diagnostic training includes didactic instruction, observation, and interview practice before being observed and evaluated for clearance by senior study members. The clinician version of the SCID-5 (κ > .70; [142]) and the exp-SADS-L (κ > 0.90 for MDD; [8, 50, 143] have shown good to excellent inter-rater reliability for both mood and other diagnoses.

#### Self-report symptom measures

At T1-T6, every 6 months, participants complete a variety of self-report symptom measures assessing mood symptoms and substance use. Depressive symptom severity is assessed using two self-report scales. First, the Beck Depression Inventory-II (BDI-II) [144] is a 21-item questionnaire that asks participants to use a 0 to 3 scale to indicate which statement best describes their feelings over the past two weeks. The BDI-II has demonstrated strong reliability and validity in community and adolescent samples [145, 146]. Second, depressive symptoms are assessed using the 10D subscale of the 10 Mania-10 Depression Scales (10M10D) [147], which are short forms with items drawn from the General Behavior Inventory [148]. The 10D scale is made up of 10 items that address key depressive symptoms (e.g., feelings of sadness and anhedonia). The 10D scale has demonstrated reliability in adolescent self-report (α > 0.9 across settings) and displays excellent convergent and discriminant validity [147].

Symptoms of hypo/mania are assessed using three measures of symptom severity. The first measure is the Altman Self-Rating Mania Scale (ASRM) [149]. This questionnaire asks participants to rate how they have been feeling for the past week on a five-point Likert scale for five items addressing key hypo/manic symptoms (elevated mood, inflated self-confidence, decreased need for sleep, excessive talkativeness, and increased activity). The ASRM has good internal consistency for a short scale (α ranging from 0.65 to 0.79) and demonstrates retest and convergent validity [149]. Second, hypo/manic symptoms are evaluated using the 10 M scale of the 10M10D. The 10 M scale is made up of 10 items that address key hypo/manic symptoms (e.g., high mood and energy). The 10 M scale has demonstrated reliability in adolescent self-report (α > 0.85 across settings) and displays excellent convergent and discriminant validity [147]. Finally, participants also complete the 10-item version of the Child Mania Rating Scale (CMRS) [150], which is an adapted version of the original 21-item measure [151]. The 10-item version utilizes the items that were statistically best at identifying hypo/mania from the full scale (e.g., periods of too much energy) and demonstrates reliability (α = 0.91) and strong discriminant validity [150].

Participants also complete a self-report measure of the frequency of drug and alcohol use, the Adolescent Alcohol and Drug Involvement Scale (AADIS) [152]. The AADIS is an adaptation of the Adolescent Drug Involvement Scale [153] that asks participants to rate how often they use various substances (e.g., alcohol, marijuana, hallucinogens) on a seven-point scale from *Never used* to *Several times per day*. The AADIS is a reliable and consistent measure [152–154], with α = 0.71 in [155].

On the day of the fMRI scan, Ps complete the 15-item Internal State Scale (ISS) [156] to assess current affect and discriminate between manic and depressive mood states [157]. The scale contains activation, well-being, perceived conflict, and depression subscales. Participants are instructed to indicate the extent to which each statement applies to them today on 5-point Likert scales (*Very Slightly or Not at All* to *Extremely*). The ISS has previously shown good convergent and discriminant validity [156, 158], and has been used with several clinical and non-clinical samples [55, 63].

### Ecological momentary assessment (EMA) procedures

At T1, T3, and T5, participants complete 7 days of EMA following an in-person session using the SEMA3 online platform [159]. During the 7-day EMA periods, in addition to completing a daily sleep diary and wearing an Actiwatch, participants complete affect, symptom, and RR ratings three times a day (morning, afternoon, evening). Both the EMA surveys and sleep diaries ask the participant to report any life events that have occurred since the last prompt. This EMA period begins the day after the in-person session, which includes the fMRI, and includes the DLMO procedure on the first evening of the EMA period. The season and day of the week that the EMA period begins are recorded as potential covariates.

At the first in-person session, participants download the SEMA3 smartphone application and receive training to understand how to respond to prompts. Participants also choose the most suitable time to receive the nightly sleep diary at their in-person visit before beginning the EMA periods (choices are 7, 8, 9, or 10 PM). Sleep diary surveys must be completed within 12 h. Generally, the EMA prompt window begins 12 h before the sleep diary survey time chosen by the participant. Participants are sent a morning, afternoon, and evening survey prompt within a 3-h window (e.g., 9AM-12PM, 1-4PM, and 5-8PM clock time), and each survey expires after an hour. In some cases in which participants’ schools strictly do not allow phone use, an alternative schedule is used. In these cases, the typical schedule is used for the weekend days in the 7-day period. For the school days, participants are sent the morning survey between 6 and 7:30 AM, the afternoon survey between 3 and 5 PM, and the evening survey between 6 PM and their chosen sleep diary time. These surveys also expire after an hour.

#### EMA assessments

The EMA surveys assess current affect, depressive symptoms, hypo/manic symptoms, RR, and life events that occurred since the previous prompt. Affect is assessed using eight emotions drawn from the circumplex model of emotion [160], which, in brief, asserts that emotions exist at various levels among two dimensions: valence (pleasant to unpleasant) and arousal (activated to deactivated). Two affective states were selected from each quadrant (excited, elated, content, calm, upset, bored, depressed, tense) and participants respond on a 5-point Likert scale (*Very slightly or not at all* to *Extremely)* the extent to which they feel that way currently. Hypo/manic symptoms are assessed with the ASRM [149] described above. Depressed mood is measured using the short-form pediatric depression scale of the PROMIS (Patient-Reported Outcomes Measurement Information System), a well-validated eight-item measure developed by the NIH using advancements in psychometrics [161].

To assess RR, participants respond to two items drawn from the WASSUP (one social and one non-social reward) from a question bank comprised of six possible items. Participants also answer two novel monetary and social reward questions: “*Right now how likely are you to accept a job that pays XX an hour?*” and “*Right now how likely are you to be friends with someone who makes you feel heard and understood XX% of the time?*”. The specific dollar amount or percentage is different at each prompt.

Last, participants are asked if any life events have occurred since completing the last EMA survey in the following categories: school, romantic, family, work, friends, money, health, legal, or other. If participants respond in the affirmative, they are asked whether the event was positive, negative, or both, and asked to briefly describe what happened.

### Potential covariates

#### Pubertal development scale

Given that RR has been shown to be associated with pubertal maturation [162], all participants complete the 5-item Pubertal Development Scale (PDS) [163]. The PDS is given every 6 months at T1-T6 for the duration of the study, or until participants have reached full maturity. The PDS includes items regarding growth in height and weight, changes in skin, changes in facial hair and voice (males), and changes in breast and menstruation (females). All items excluding menstruation are scored on a 4-point scale ranging from 1 (*no development*) to 4 (*full development*). The menstruation item is scored as a 1 (*I have not yet begun to menstruate*) or a 4 (*I have begun to menstruate*). Scores are averaged across items resulting in a possible final score of 1 through 4, with a higher score indicating greater pubertal development. The PDS displays good reliability (*a* = 0.77) [163] and satisfactory convergent validity with physicians’ ratings (*r’s* ranging from 0.61 to 0.67; [164–167]). The original source of the description of the PDS may be found in Alloy et al. [91].

#### Handedness

Participant handedness is assessed at T1 using the Chapman Handedness Scale [168]. This scale includes 13 items that ask the participant to report which hand they use for various activities (e.g., writing). Each item is scored from 1–3 (1 indicates right hand, 2 is either hand, and 3 is left hand). A score of 13 indicates that an individual is completely right-handed whereas a score of 36 indicates complete left-handedness. Individuals who score between 13 and 17 are considered right-handed, those who score between 18 and 32 ambidextrous, and those who score between 33 and 39 left-handed. The scale demonstrates high internal consistency (α = 0.96) as well as retest reliability.

#### Medication

Participants are probed for medication usage in three areas from the SCID-5 interview at each time point: dosage, start date, and end date. We do not expect high rates of medication use at T1, given that we are excluding adolescents with past BSD. To increase representativeness and generalizability of project findings, we will not exclude adolescents who are taking psychotropic medications. Instead, analyses will control for use of medications and we also will conduct sensitivity analyses by excluding the data of participants who are taking medications that may affect cortico-striatal function. If warranted, the presence of other mental disorders and family history of mood disorders also will be controlled.

## Discussion

Future results from this study should be considered in light of several limitations. First, with the exception of the Positive Valence Systems Scale (PVSS), which assesses multiple domains of reward processing, our measures of RR primarily focus on reward anticipation and consumption and do not comprehensively assess other components of reward function, such as reward learning or effort expenditure for reward. Unfortunately, these additional assessments were not possible within the scope of budget limitations but would be important directions for future work on reward-circadian mechanisms involved in bipolar spectrum disorders. Second, ideally, dim light melatonin onset time would be assessed over several days at T1, T3, and T5 to obtain a more stable estimate of participants’ circadian phase; however, this was not feasible due to budgetary and practical limitations. Third, we assess circadian rhythms in the context of naturally occurring life events that may disrupt social rhythms, but do not measure circadian rhythm disruption in response to a challenge (e.g., experimental manipulation of light exposure or daily schedule disruption).

These limitations notwithstanding, the findings from Project CREST hold promise for advancing understanding of vulnerabilities and mechanisms involved in the emergence of bipolar spectrum disorders in adolescence. Although prior research has separately examined the relationship of reward responsiveness and BSD and social and circadian rhythms and BSD, no studies have taken the integrative, multi-organ, perspective that we take in this study. Drawing on research highlighting bidirectional influences between the reward and circadian systems (e.g., [24, 69], we propose a novel integrative Reward Circadian Rhythm (RCR) model, which predicts that dysregulated signaling between reward neural circuitry and circadian rhythms is a joint vulnerability for bipolar symptoms [24]. In line with the Research Domain Criteria and Goals 1 and 2 of the NIMH Strategic Plan, this project is the first test of the relationship between RR in both monetary and social domains, social and circadian rhythm disruption, and first onset of BSD and increases in bipolar symptoms during adolescence, an “age of risk” for development of BSD. Our high-risk, longitudinal design with multiple time points allows us to assess whether abnormalities in reward-circadian signaling predate the onset of first BSD, reflecting a preexisting vulnerability, or emerge as a consequence of the illness. This is important for understanding etiological pathways to bipolar conditions and identifying biobehavioral markers of risk. Project CREST also is innovative in utilizing a combined micro- (EMA) and macro-longitudinal design that allows assessment of RR and circadian function and their prediction of bipolar symptoms with ecological validity, temporal precision, and sensitivity in ambulatory community-dwelling adolescents. Identifying reward-circadian pathways in the emergence of BSD and bipolar symptoms also could facilitate “a next generation” of behavioral and biological interventions that target reward-to-circadian and/or circadian-to-reward signaling to mitigate circadian and RR abnormalities in order to treat, and ideally prevent, BSDs and their lifelong negative consequences.

### Supplementary Information

**Additional file 1.** STROBE Statement for Project CREST—checklist of items that should be included in reports of observational studies.

## Data Availability

Data from this study will be available on the NIMH National Data Archive (Collection #C4089).

## Abbreviations

10M10D: 10 Mania 10 Depression Scales
AADIS: Adolescent Alcohol and Drug Involvement Scale
ASRM: Altman Self-Rating Mania Scale
BDI-II: Beck Depression Inventory-II
BIS/BAS: Behavioral Inhibition System/Behavioral Activation System Scales
BSDs: Bipolar Spectrum Disorders
CARROT: Card Arranging Reward Responsivity Objective Test
CLES: Children’s Life Events Scale
CMRS: Child Mania Rating Scale
CREST: Circadian, Reward, and Emotion Systems in Teens
DLMO: Dim Light Melatonin Onset
DSM: Diagnostic and Statistical Manual of Mental Disorders
EMA: Ecological Momentary Assessment
fMRI: Functional Magnetic Resonance Imaging
ISS: Internal State Scale
LEI: Life Events Interview
LES: Life Events Scale
MEQ: Morningness-Eveningness Questionnaire
MID: Monetary Incentive Delay Task
OFC: Orbitofrontal Cortex
PDS: Pubertal Development Scale
PPI: Psychophysiological Integration
PROMIS: Patient-Reported Outcomes Measurement Information System
PSQI: Pittsburgh Sleep Quality Index
PVSS: Positive Valence Systems Scale
RCR: Reward Circadian Rhythm Model
ROI: Regions of interest
RPAS: Responses to Positive Affect Scale
RR: Reward responsiveness
RRS: Ruminative Responses Scale
SADS-L: Schedule for Affective Disorders and Schizophrenia-Lifetime
SCID-5: Structured Clinical Interview for DSM-5
SCN: Suprachiasmatic Nucleus
SOL: Sleep Onset Latency
SPSRQ: Sensitivity to Punishment Sensitivity to Reward Questionnaire
SRD: Social Rhythm Disruption
SRM-T: Social Rhythm Metric-Trait
T: Time
vmPFC: Ventromedial Prefrontal Cortex
VS: Ventral Striatum
WASO: Wake After Sleep Onset
WASSUP: Willingly Assumed Set of Statistically Unlikely Pursuits
